# The case for an international severity of illness scoring system

**DOI:** 10.62675/2965-2774.20250293

**Published:** 2025-02-28

**Authors:** Alexander Tracy, Jorge Ibrain Figueira Salluh, Eirik Alnes Buanes, Dave A Dongelmans, Stefano Finazzi, Bharath Kumar Tirupakuzhi Vijayaraghavan, Nazir Lone, David Pilcher, Matti Reinikainen, Menbeu Sultan, David Thomson, Wangari Waweru-Siika, Rashan Haniffa, Abigail Beane

**Affiliations:** 1 Oxford University Hospitals NHS Trust - Oxford John Radcliffe Hospital Nuffield Department of Anaesthesia Oxford United Kingdom Nuffield Department of Anaesthesia, John Radcliffe Hospital, Oxford University Hospitals NHS Trust - Oxford, United Kingdom.; 2 Instituto D’Or de Pesquisa e Ensino Department of Critical Care Rio de Janeiro RJ Brazil Department of Critical Care, Instituto D’Or de Pesquisa e Ensino - Rio de Janeiro (RJ), Brazil.; 3 Haukeland University Hospital Department of Anaesthesia and Intensive Care Bergen Norway Department of Anaesthesia and Intensive Care, Haukeland University Hospital - Bergen, Norway.; 4 University of Amsterdam Amsterdam UMC Department of Intensive Care Medicine Amsterdam Netherlands Department of Intensive Care Medicine, Amsterdam UMC, University of Amsterdam - Amsterdam, The Netherlands.; 5 Mario Negri Institute for Pharmacological Research IRCCS Laboratory of Clinical Data Science Department of Medical Epidemiology Lombardia Italy Laboratory of Clinical Data Science, Department of Medical Epidemiology, Mario Negri Institute for Pharmacological Research IRCCS - Lombardia, Italy; 6 Apollo Hospitals Department of Critical Care Medicine Chennai India Department of Critical Care Medicine, Apollo Hospitals - Chennai, India.; 7 University of Edinburgh Usher Institute Edinburgh United Kingdom Usher Institute, University of Edinburgh - Edinburgh, United Kingdom.; 8 Alfred Health Department of Intensive Care Melbourne Australia Department of Intensive Care, Alfred Health, Melbourne, VIC 3004 – Melbourne, Australia.; 9 Kuopio University Hospital Department of Anaesthesiology and Intensive Care Kuopio Finland Department of Anaesthesiology and Intensive Care, Kuopio University Hospital - Kuopio, Finland.; 10 St. Paul's Hospital Millenium Medical College Addis Ababa Ethiopia St. Paul's Hospital Millenium Medical College - Addis Ababa, Ethiopia.; 11 Groote Schuur Hospital and University of Cape Town Division of Critical Care Department of Anaesthesia and Peri-operative Medicine Western Cape South Africa Division of Critical Care, Department of Anaesthesia and Peri-operative Medicine, Groote Schuur Hospital and University of Cape Town - Western Cape, South Africa.; 12 Aga Khan University Department of Anaesthesia Nairobi Kenya Department of Anaesthesia, Aga Khan University – Nairobi, Kenya.; 13 University of Edinburgh Centre for Inflammation Research Edinburgh United Kingdom Centre for Inflammation Research, University of Edinburgh - Edinburgh, United Kingdom.

## INTRODUCTION

Severity of illness scores in the critical care context have evolved to serve multiple functions. These scores enable risk-adjusted outcomes to be benchmarked for the assessment of intensive care unit (ICU) performance, inform resource allocation, and enable the characterization of disease severity. Numerous illness severity scores have been developed to optimize calibration at the national level, but few studies have examined the international application of such scores.^(1)^

The COVID-19 pandemic demonstrated the value of benchmarking outcomes across heterogeneous populations and across various health care systems. Severity of illness scores were widely used to describe trial populations, assess treatment effects and evaluate the quality of care during the pandemic. Similar use cases apply to other priorities for international critical care research and quality improvement, such as improving outcomes following traumatic injuries and expanding access to complex medical, surgical and obstetric care.^(2,3)^ Therefore, this article argues that the development of an international illness severity score is an urgent priority for critical care research.

## CURRENT LIMITATIONS TO THE INTERNATIONAL USE OF ILLNESS SEVERITY SCORES

The international use of current illness severity scores poses several challenges. Firstly, such scores are often constructed in a way that limits their global relevance. For example, many scores incorporate diseases and comorbidities that are predominant in critically ill patients from high- and middle-income countries. Age categories also reflect life expectancies and outcomes in higher-income contexts. Score performance may be further impacted by geographical differences in the age profile of patients admitted to ICUs.^(4)^

Secondly, many illness severity scores incorporate variables, such as laboratory investigation results, that are not universally available in all settings. Therefore, there is a high prevalence of missing data when the scores are used in low-income countries.^(5-7)^ This may occur because certain investigations are not readily available or because of differences in decision-making and prioritization by clinicians in different settings. Such variations in clinical practice are not captured by illness severity scores that incorporate these variables. Efforts to address this limitation have been hindered by the inadequate reporting of missing values in studies that examine illness severity scores in lower- and middle-income countries.^(7)^

Furthermore, existing illness severity scores fail to account for differences between health care contexts. Health care system design, case mix and the availability of treatment modalities are all factors that are likely to influence score performance. Importantly, ICUs serve different purposes in different health care systems, leading to marked variations in pre- and post-ICU care.^(8)^

Despite these limitations, scores developed using regional datasets are used to stratify patients in large clinical trials. This practice is problematic because the results generated from such trials are frequently applied to the global population. Moreover, this hinders the synthesis of disease severity-stratified data for systematic reviews and meta-analyses.

## OPPORTUNITIES PRESENTED BY AN INTERNATIONALLY APPLICABLE ILLNESS SEVERITY SCORE

The identification of an internationally applicable illness severity score would afford many opportunities to advance global critical care research and public health action (Figure 1). Observational research would benefit from epidemiological studies that span a broad range of populations and health care systems, as these studies would improve the understanding of specific conditions that occur globally. An international illness severity score would also facilitate international benchmarking of ICU outcomes. This would be especially beneficial in countries with limited ICU capacity or without established benchmarking programs, and would facilitate the identification of variations in ICU performance between health care systems.

**Figure:**
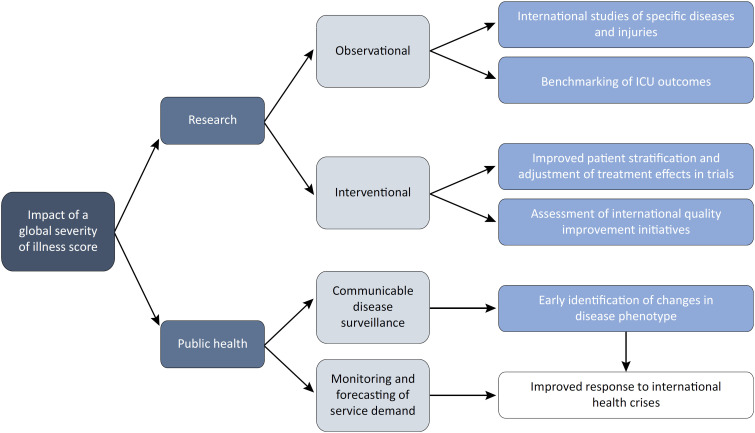
Opportunities presented by a global severity of illness score.

ICU - intensive care unit.

Interventional research would benefit from the improved design of clinical trials so that their findings can be translated to a global context. Multinational trial recruitment may be facilitated, thus increasing the global representativeness of trial populations. Additionally, an international score would aid in the assessment of quality improvement initiatives for both patients with specific diseases and the broader critical care population.

Potential public health benefits include improved surveillance of critical illness secondary to communicable diseases, such as monitoring changes in disease phenotype and severity over time. Moreover, a valid score could be used to aid in tracking and forecasting ICU service demands. This approach would be particularly valuable when demands are influenced by international events such as pandemics, conflicts and natural disasters.

## DEVELOPMENT OF EXISTING INTERNATIONAL SEVERITY OF ILLNESS SCORES

These opportunities have motivated the development of regional customizations of commonly used models, such as the SAPS 3 and APACHE IV scores.^(9)^ An alternative approach has been to develop and validate scores, such as the Global Open Source Severity of Illness Score (GOSSIS), using international datasets.^(10)^ However, neither strategy has addressed the unavailability of certain data in lower-income settings. Furthermore, the resulting scores have not been validated across a full range of socioeconomic contexts.

Some ‘simplified’ scores, such as the severity assessment score (SEVERITAS), the Simplified Mortality Score for the ICU (SMS-ICU), the Universal Vital Assessment (UVA) and the Tropical Intensive Care (TropICS) scores, address data unavailability by using a small number of core variables.^(6,11-13)^ However, it is still necessary to evaluate score performance in globally representative populations spanning multiple continents and economic conditions. The global growth of critical care registries provides an ideal opportunity to undertake such work in a real-world setting. Collaborative initiatives to link national critical care registries could play a key role in this research.^(14)^

An international score would likely be inferior to existing locally calibrated scores when applied to individual countries. Therefore, an international score would not replace scores that are currently used for local research or national benchmarking. Instead, an international score provides a common language for describing illness severity in the context of international collaboration.

## CONCLUSION

International collaboration is essential for the development of informative critical care research and effective public health action. The identification of an international severity of illness score would yield significant opportunities for research, quality improvement and service development at the global level.

An international severity of illness score should use a small dataset consisting of variables routinely available in ICUs worldwide. Furthermore, it should be validated using real-world data from critical care registries across the full range of geographical regions. A suitable score may be constructed *de novo* or may result from customization of an existing model. The performance of an international score may potentially be improved by the inclusion of socioeconomic, geographical, and health care system-related variables.

There is increasing interest in addressing the global challenges faced by ICUs, supported by the expansion of real-world critical care datasets and the use of common data definitions. Therefore, it is now time to prioritize the development of an international severity of illness scoring system.
